# Controllable Preparation and Enhancement Mechanism of Al_2_O_3_ Nanomaterial-Modified Ultrafine Cement Composite Grouting Materials

**DOI:** 10.3390/nano16160987

**Published:** 2026-08-10

**Authors:** Xiang Cheng, Chaoyu Tian, Yanfen Wang, Guangming Zhao, Gangzheng Liu, Yingming Li, Xiangrui Meng, Lianqin Ni

**Affiliations:** 1 State Key Laboratory of Digital Intelligent Technology for Unmanned Coal Mining, Anhui University of Science and Technology, Huainan 232001, China; cxaust@aust.edu.cn (X.C.); 2024202160@aust.edu.cn (C.T.); 2020116@aust.edu.cn (L.N.); 2 School of Materials Science and Engineering, Anhui University of Science and Technology, Huainan 232001, China; 2024201076@aust.edu.cn; 3 School of Mining Engineering, Anhui University of Science and Technology, Huainan 232001, China; guangmingzhao@163.com (G.Z.); yingmingli@aust.edu.cn (Y.L.); xrmeng@aust.edu.cn (X.M.)

**Keywords:** Portland cement, mechanical properties, workability, microstructural evolution, mine reinforcement

## Abstract

Using ultrafine silicate cement as the cementitious material, and admixtures such as expansion agent, rapid-setting agent and water reducer as additives, a new type of composite grouting material with high early strength and high toughness was obtained by modification with nano-Al_2_O_3_ (NA). The influence of NA content on the mechanical properties, flowability, bleeding behavior, setting time, volume shrinkage, and microstructure was investigated for composite grouting materials. The results show that, as the NA content increases, the flowability of the paste decreases, the bleeding rate reduces, and the setting time increases first and then decreases. Appropriate NA can effectively improve the mechanical strength of the composite grouting material. Especially at the condition of 3% NA, the composite grouting material reached the highest early compressive strength and toughness. Compared with the control group, the compressive strength of the specimen increases by 39.54% and 6.83% respectively at 1 d and 21 d, and the flexural strength increased by 55.41% at 1 d. XRD, FTIR, SEM and hydration heat analysis confirm that the NA can promote the early hydration heat of ultrafine cement, and shorten the induction period. Moreover, more C-A-H gel products will be generated by consuming Ca(OH)_2_ with active NA, leading to an improvement in the matrix compactness. Such an outstanding mechanical property can be mainly attributed to the triple coupling mechanism of the hydration regulation, microstructure and filling effect of superfine cement by nano-Al_2_O_3_ in combination with multi-component admixtures.

## 1. Introduction

With the gradual complexity of geological conditions in coal mines, such as soft and fractured surrounding rock, high ground stress, and large deformation of surrounding rock, significant challenges have been brought to the safe and efficient mining of coal mines. Anchor-grouting support technology plays a key role in controlling roadway surrounding rock. Especially, to achieve full-length anchoring support, grouting materials are generally required to have adequate flowability, low bleeding, slight expansion, appropriate setting time, and sufficient mechanical strength [1,2,3,4]. In the grouting material system, cement-based materials have attracted significant attention due to their low cost and good durability, and the wide availability of raw materials. However, in mine grouting reinforcement, conventional cement grouts often encounter problems such as bleeding, slow early strength development, and insufficient penetration into fine fractures [5,6,7]. Consequently, targeted modification of the physical and chemical properties of cement-based grouts is required to achieve the desired performance.

Among nano-oxides, Al_2_O_3_-based structures have attracted attention because their phase composition, surface chemistry, morphology, and pore structure are tunable. Nano-Al_2_O_3_ has been investigated in construction, ceramic, and biomedical materials [8,9,10]. In cementitious systems, nano-Al_2_O_3_ can regulate cement hydration through particle filling, heterogeneous nucleation, and chemical interactions, thereby improving material performance. Al_2_O_3_ powders can be prepared by solgel, precipitation, hydrothermal, and combustion methods. Recent studies on in situ growth and carrier-assisted modification further show that preparation and dispersion markedly affect rheology, hydration, and strength development in cementitious systems [11,12,13]. Li et al. [14] found that an appropriate nano-Al_2_O_3_ dosage improves concrete strength and resistance to chloride penetration, whereas higher dosages reduce workability and increase porosity and water absorption. Joshaghani et al. [15] reported that high dosages or particle agglomeration weaken the strengthening effect. Crystal phase can also affect long-term performance. Yu et al. [16] found that both α- and γ-Al_2_O_3_ accelerate strength degradation and expansion under sulfate exposure, with γ-Al_2_O_3_ causing more pronounced long-term deterioration. Therefore, the effects of Al_2_O_3_ in cementitious systems cannot be evaluated solely on the basis of dosage. Crystal phase, material composition, dispersion state, and target properties must also be considered.

To further clarify the differences between previous studies and the present work, Table 1 compares representative studies in terms of material system, Al_2_O_3_ characteristics, evaluation scope, and main findings.

As shown in Table 1, the Al_2_O_3_ crystal phases, dosage ranges, and cementitious systems used in different studies vary considerably, and there is no consistent parameter range for their beneficial effects. Existing studies mainly focus on ordinary cement pastes, mortars, or concrete and generally emphasize only one aspect, such as hydration mechanisms, mechanical properties, or durability. Research directly addressing ultrafine Portland cement-based grouting materials remains relatively limited, and existing studies generally evaluate only some performance indicators. Therefore, in a multi-component ultrafine cement grouting system containing an accelerator, expansive agent, and superplasticizer, the combined effects of Al_2_O_3_ dosage on workability, mechanical properties, and hydration evolution remain insufficiently understood.

Building on previous studies of nano-Al_2_O_3_-modified cementitious materials, this study extends the investigation to a multi-component ultrafine cement grout containing fixed dosages of accelerator, expansive agent, and superplasticizer, with the aim of determining how the nano-Al_2_O_3_ dosage governs its performance evolution. The specific objectives are to clarify the dosage-dependent changes in flowability, setting behavior, bleeding, volume stability, and mechanical strength, to quantify the relationships among these macroscopic properties, and to relate these changes to hydration kinetics and microstructural evolution. On this basis, the study seeks to identify a suitable nano-Al_2_O_3_ dosage within the investigated range and clarify its strengthening mechanism in the multi-component ultrafine cement grouting system.

## 2. Experimental Method

### 2.1. Raw Material

**Figure 1:**
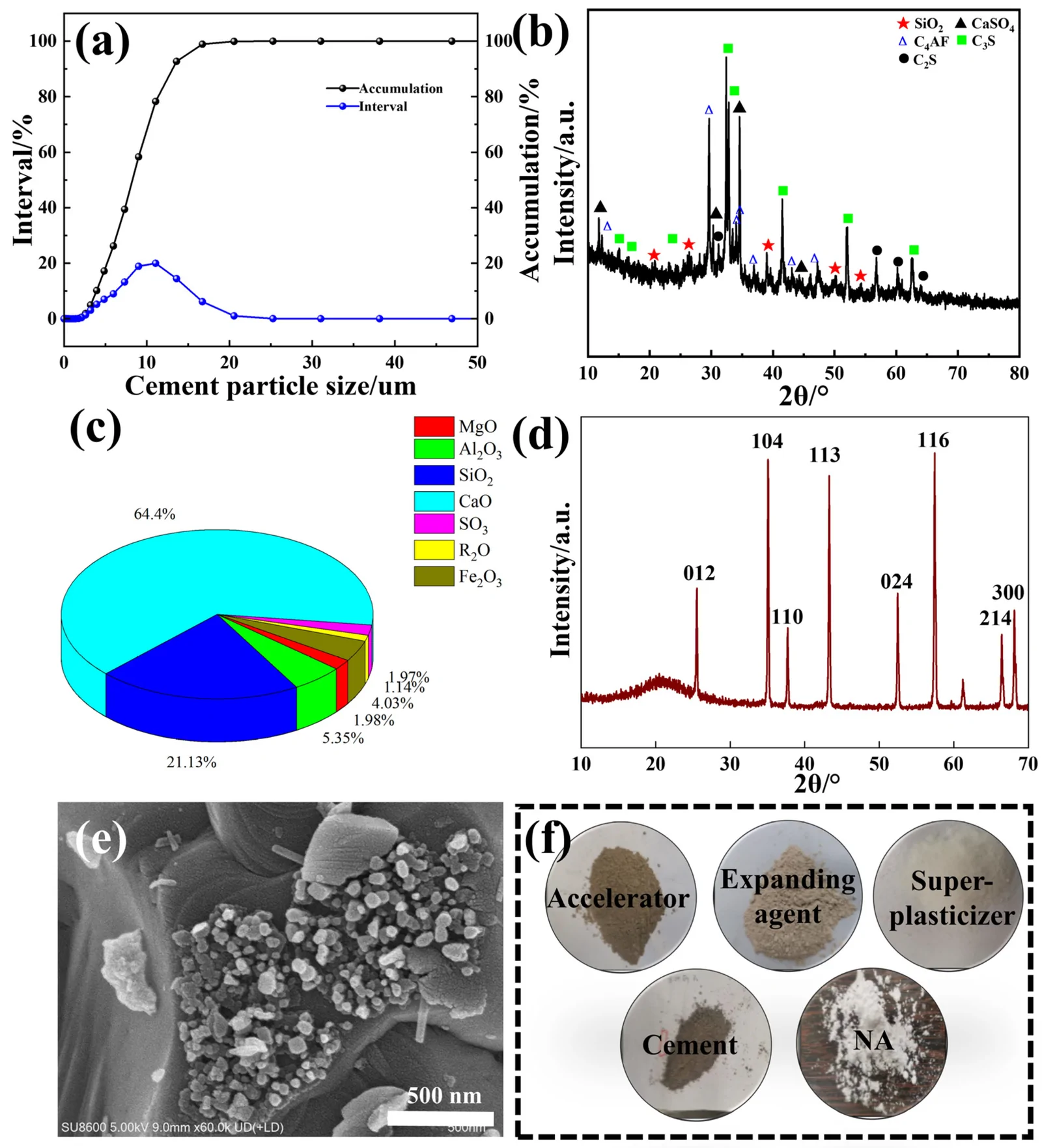
Morphological characteristics and phases of raw materials: (**a**) particle size distribution; (**b**) XRD pattern and (**c**) chemical composition of ultrafine cement; (**d**) XRD pattern and (**e**) SEM image of NA particles; (**f**) image of all raw materials.

Ultrafine cement, produced by Zhejiang Sanshi Special Materials Co., Ltd. (Jinhua, China), is a type of silicate cement with an average particle size of 10 μm (Figure 1a). The XRD pattern and main components are shown in Figure 1b,c. The admixtures include polycarboxylate superplasticizer, expansion agent (Henan Xinmi Mingzhu Waterproof and Anticorrosive Materials Co., Ltd., Xinmi, China), and accelerator (Huaibei Xinda New Building Materials Co., Ltd., Huaibei, China). Nano-Al_2_O_3_ (NA) was purchased from Qinghe County Zhongzhou Alloy Materials Co., Ltd. (Xingtai, China), and the powder had a density of 3.9 g/cm^3^. The XRD pattern shown in Figure 1d confirmed that the powder was mainly composed of α-Al_2_O_3_. Based on the fitted peak widths of eight diffraction peaks, its apparent coherent diffraction domain size was estimated to be approximately 59 nm using the Scherrer equation. The SEM image in Figure 1e reveals abundant nanoscale Al_2_O_3_ particles attached to or clustered around micrometer-scale structures, indicating a hierarchical morphology. Figure 1f shows photographs of the raw materials used for the grouting material.

### 2.2. Mix Ratio and Sample Preparation

The mix proportions were designed based on previous experimental results, the literature data, and preliminary tests, as shown in Table 2 [20,21,22]. Ultrafine cement, NA, accelerator, expansive agent, and superplasticizer were weighed according to the specified mass ratios and added into a cement mortar mixer. The mixture was dry-blended at a low speed (140 ± 5 r/min) for 2 min. Subsequently, water was added at a water–cement ratio of 0.35, and the mixture was continuously stirred at a high speed (285 ± 10 r/min) for 120 s. After the mixing was completed, the slurry was allowed to stand for 30 s to eliminate air bubbles, and then it was blended at a low speed for 30 s. This process resulted in ultrafine cement composite slurries with different NA. Subsequently, their working performance, mechanical properties, and microstructure were characterized. Each group of tests was repeated three times and the average value was taken.

### 2.3. Test Methods

#### 2.3.1. Setting Time, Bleeding Rate and Flowability

According to the “Test Methods for Standard Consistency Water Content, Setting Time and Soundness of Cement” (GB/T 1346-2011), the setting time of the grouting material was tested by a Vicat apparatus (Zhejiang Guangnian Zhixin Instrument Co., Ltd., Shaoxing, China). A total of 100 mL of the grouting material was poured into a graduated cylinder, and left to stand for 60 min. The corresponding scale at the interface between the upper clear water and lower slurry was recorded to calculate the bleeding rate. The grouting material was poured into a conical cylindrical mold with dimensions of 60 × 70 × 100 mm^3^ (height × inner diameter of upper opening × inner diameter of lower opening), filled to the brim, and leveled to make the slurry level with the upper opening of the mold. Then, it was lifted steadily to allow the slurry to flow freely without disturbance until it stopped forming a circular slurry. The diameter of the circle was measured, which was the flowability.

#### 2.3.2. Mechanical Strength and Expansion Rate

The prepared NA-modified ultrafine cement composite slurry was poured into 40 × 40 × 160 mm molds. After vibration and leveling, the specimens were kept in a curing box for 24 h prior to demold. Subsequently, the samples were cured at a temperature of 20 ± 2 °C and a relative humidity of 99% for different curing ages (1 d ± 30 min, 7 d ± 2 h, 14 d ± 2 h, 21 d ± 2 h). Various properties of the grouting materials were tested as required by the experiments. The volumetric expansion rate at age t was calculated as E_t_ = [(V_t_ − V_0_)/V_0_] × 100%, where V_0_ is the initial specimen volume and V_t_ is the measured specimen volume at age t. The compressive strength of the specimens was tested by an automatic cement constant-stress testing machine (YES-300, Shanghai Yingsong Mining and Industrial Equipment Instrument Co., Ltd., Shanghai, China). The flexural strength was tested using an electric cement flexural strength tester (DKZ-5000, Xiyi Building Materials Instrument Factory, Wuxi, China).

#### 2.3.3. Microscopic Characterization

**Figure 2:**
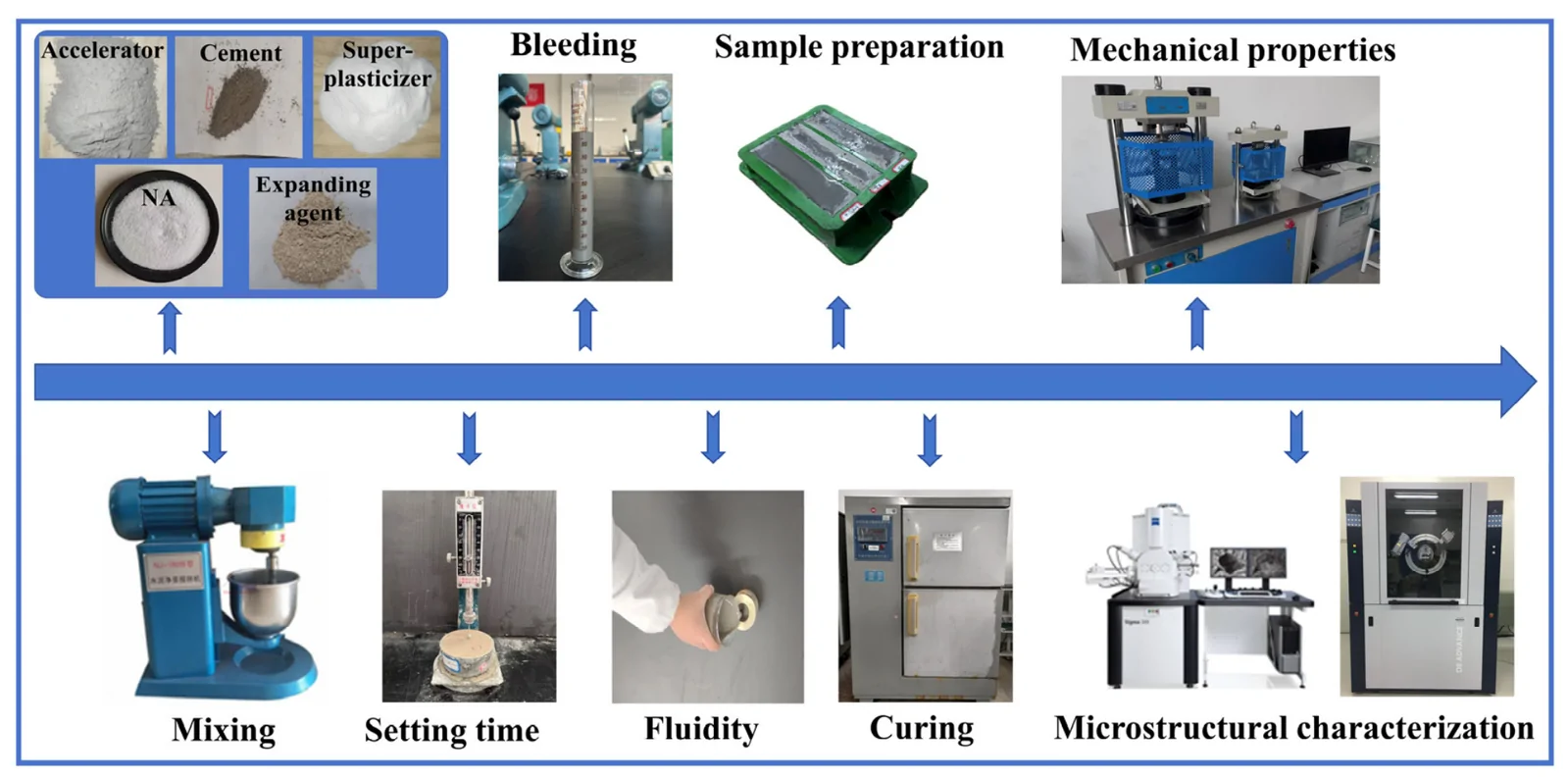
Schematic diagram of the specimen preparation and testing process.

The phase composition of the hardened specimens was analyzed using an X-ray diffractometer (XRD, SmartLab, Rigaku, Tokyo, Japan) with Cu Kα radiation (λ = 1.5406 Å) over a 2θ range of 5–80° at a scanning rate of 5°/min. The microstructural morphology of the specimens was examined using a scanning electron microscope (SEM, S-3000N, Hitachi, Tokyo, Japan). The functional groups were characterized using a Fourier transform infrared spectrometer (FTIR, Nicolet iS50, Thermo Fisher Scientific, Waltham, MA, USA) over a wavenumber range of 500–4000 cm^−1^. The hydration heat evolution was monitored using an isothermal calorimeter (TAM Air, TA Instruments, Stockholm, Sweden). Figure 2 shows the schematic diagram of the specimen preparation and testing procedures.

## 3. Results and Discussion

### 3.1. Setting Time

**Figure 3:**
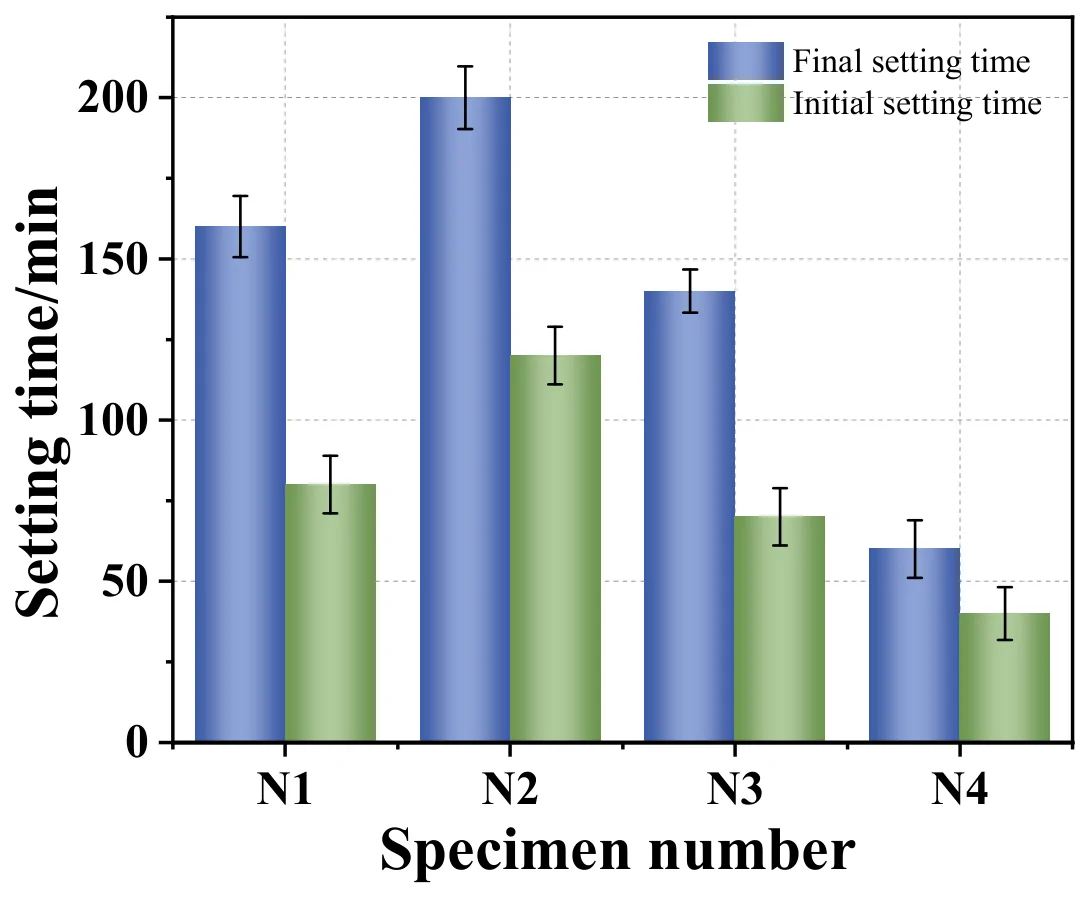
Setting time of NA-modified superfine cement composite grouting materials.

Figure 3 shows the setting time of the composite grouting materials modified with different NA. When the NA content is 1%, 3%, and 5%, the initial setting times of the composite grouting material are 200, 140, and 60 min, respectively, representing increases of 25% and decreases of 12.5% and 62.5% compared to N1. The final setting time exhibits a similar change trend to the initial setting time. These results indicate that, as the NA dosage increases, the setting time of the composite grouting material first increases and then decreases, consistent with the findings reported by Zhang et al. [23]. This is because fine Al_2_O_3_ nanoparticles can fill capillary pores in the cement paste, reducing ion migration rates [24,25,26]. As the NA content further increases, the setting time continues to decrease. This may be related to the increased number of particle surfaces that provide nucleation sites and promote the early formation of hydration products, thereby accelerating the setting process.

### 3.2. Flowability

**Figure 4:**
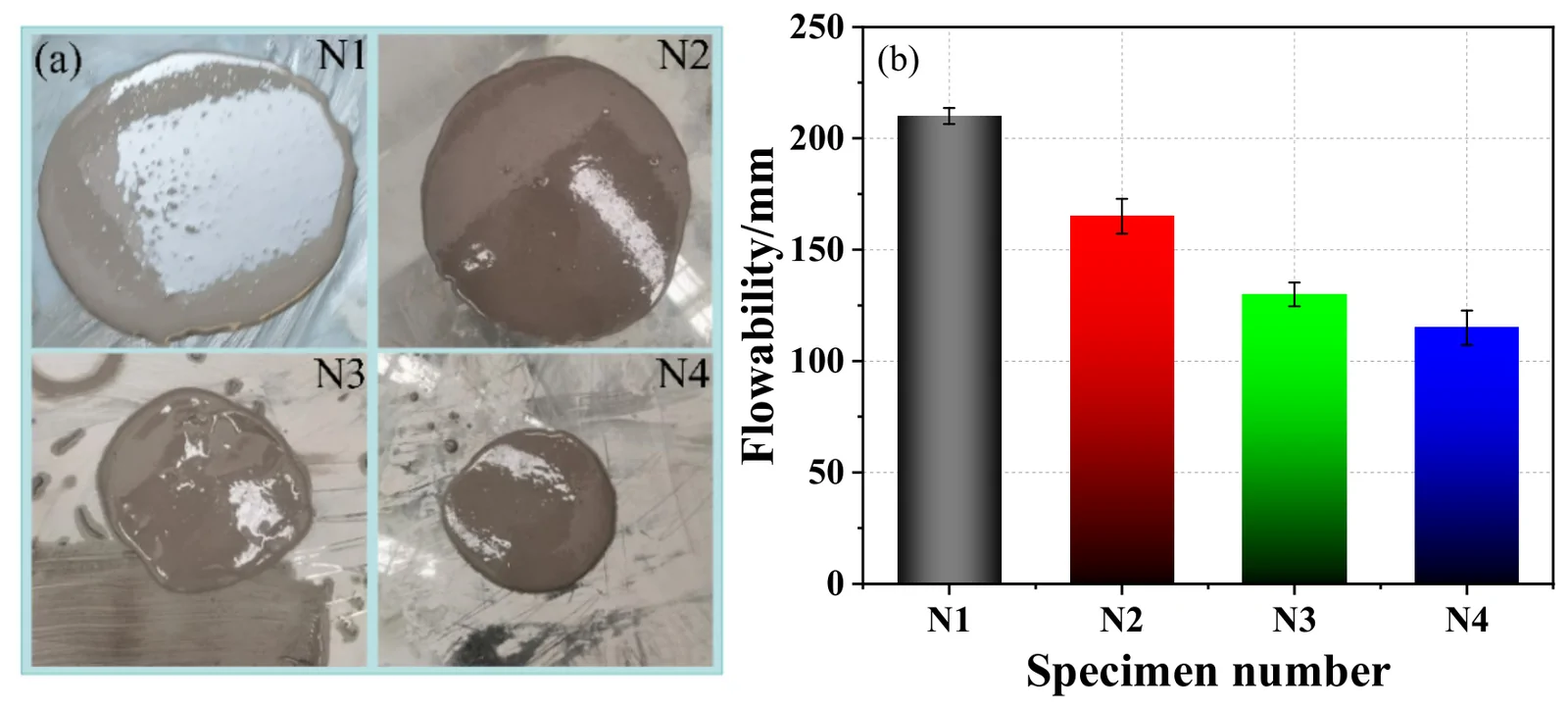
Flowability of NA-modified ultrafine cement composite grouting materials: (**a**) spread morphologies; (**b**) flowability values.

Figure 4 shows the flowability of ultrafine cement composite grouting materials modified with different NA. The flowabilities of N1 to N4 are 210 mm, 165 mm, 130 mm, and 116 mm, respectively. When the NA dosage is increased to 1%, 3%, and 5%, the flowability decreases by 21.43%, 38.10%, and 44.76% compared to N1, respectively. This indicates that, as the NA dosage increases, the flowability continuously decreases, and the rate of decline gradually intensifies. Roussel and Coussot et al. [27] established a correlation between yield stress and flowability for cone flow tests, as shown in Equation (1):

(1) $$\tau_{y}=\frac{{225\rho gV}^{2}}{{128\pi }^{2}R^{5}}$$

where τ_y_ is the yield stress, g is gravitational acceleration (m/s^2^), V is the volume of the cone (m^3^), R is the flow diameter (m), and ρ is density (kg/m^3^). According to this equation, the yield stresses of N1 to N4 are calculated to be 2.01 Pa, 6.71 Pa, 22.12 Pa, and 24.79 Pa, respectively. As the NA content increases, the yield stress of the composite slurry continues to rise. It accelerates the formation of hydration products, which coalesce into a denser microstructure, thereby markedly enhancing the slurry’s shear resistance and yield performance.

### 3.3. Bleeding Rate

**Figure 5:**
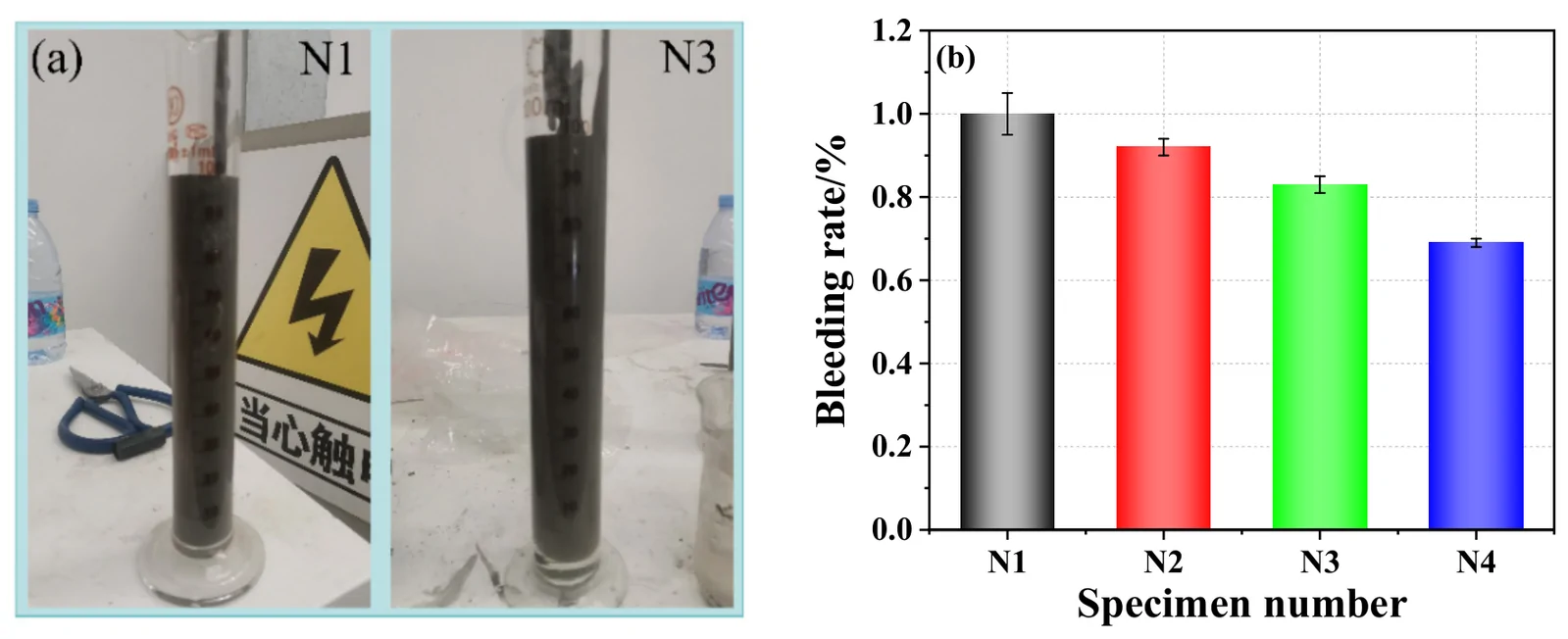
Bleeding rate of NA-modified ultrafine cement composite grouting materials: (**a**) bleeding photographs; (**b**) bleeding rate.

Figure 5 shows the bleeding rate of ultrafine cement composite grouting material with different NA. The bleeding rates for N1 to N4 are 1.0%, 0.92%, 0.83%, and 0.69%, respectively. As the NA content increases, the bleeding rate of the slurry continuously decreases, indicating an inverse correlation be-tween them. This trend may be associated with the increased fine-particle content and stronger particle interactions in the fresh slurry, which enhance slurry cohesion and hinder the upward migration and separation of water. The increased yield stress and accelerated early hydration observed at higher NA dosages may also contribute to the improved resistance to bleeding.

### 3.4. Expansion Rate

**Figure 6:**
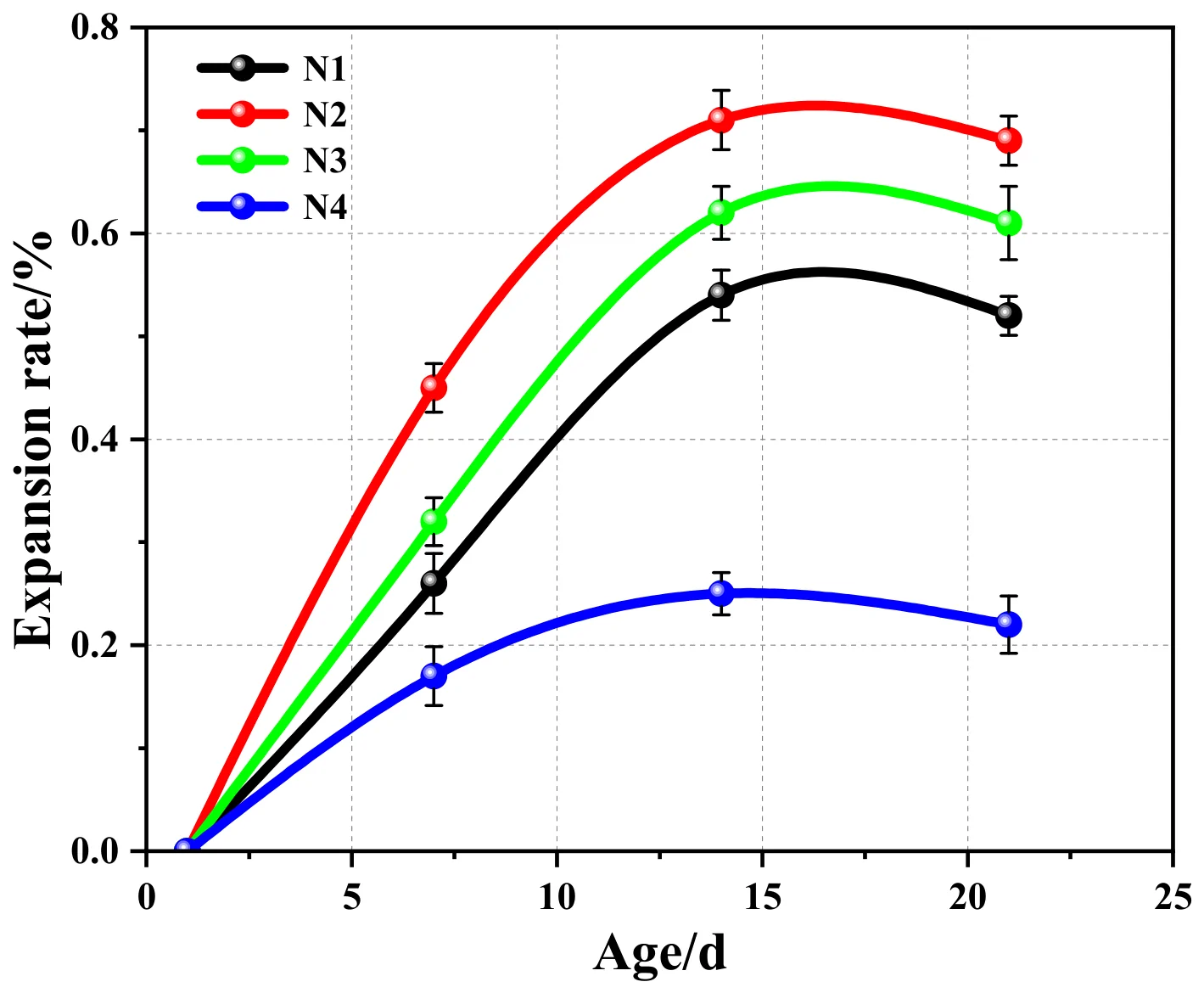
Expansion coefficient of NA-modified superfine cement composite grouting materials.

The volume change in cement slurry is primarily attributed to water reabsorption, the formation of expansive hydration products, and expansion caused by hydration heat [28,29,30], along with autogenous shrinkage and thermal shrinkage associated with hydration. Figure 6 shows the expansion rates of NA-modified ultrafine cement composite grouting materials. It can be seen that the volume expansion rate shows a trend of increasing initially and then gradually stabilizing with the duration of curing. After 21 d of curing, the expansion rates for N1 to N4 are 0.52%, 0.69%, 0.61%, and 0.22%, respectively. It is generally accepted that autogenous shrinkage is related to the water-to-cement ratio and setting time [31,32]. To counteract autogenous shrinkage, the observed expansion may result from rapid early hydration heat generation, combined with strength development that resists shrinkage strain induced by internal hydration. Additionally, a certain amount of Al_2_O_3_ doping can fill pores in the surrounding slurry, thereby reducing shrinkage strain and increasing supersaturation of silicates in the cementitious solution. However, when the dosage continues to increase, the surface tension of Al_2_O_3_ induces agglomeration, weakening this effect.

### 3.5. Mechanical Properties

**Figure 7:**
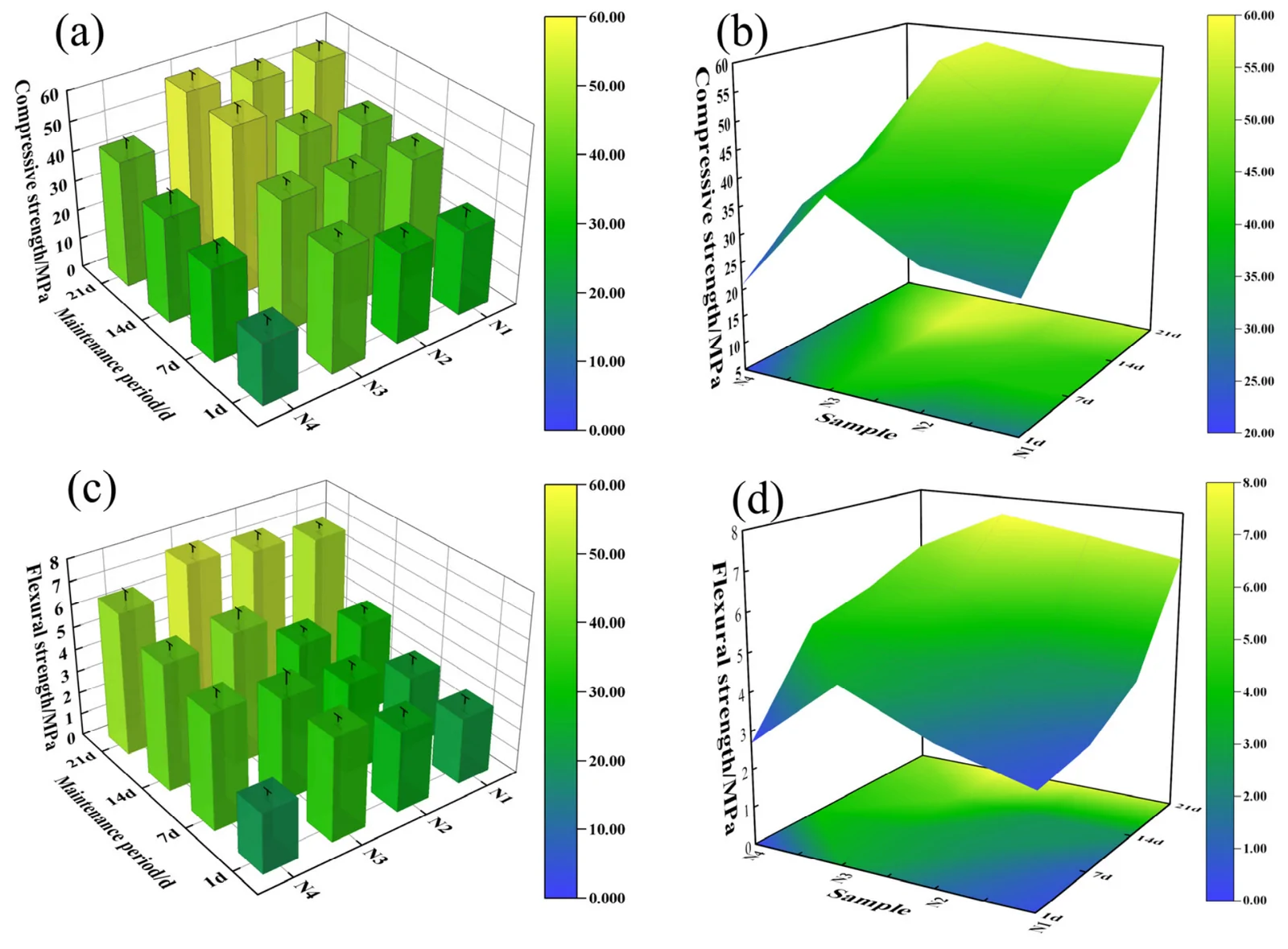
(**a**) Compressive and (**c**) flexural strength of NA-modified superfine cement composite grouting materials at various ages; (**b**,**d**) their corresponding 3D contour distribution as functions of age and NA dosage.

Figure 7 shows the mechanical strength of different NA-modified ultrafine cement composite grouting materials at various curing ages, along with their corresponding three-dimensional contour distributions. As shown in Figure 7a, the compressive strength of the solidified specimens increases initially and then decreases at different hydration stages as the NA particle content increases. The optimal compressive strength is achieved when the dosage of NA is 3%. At 1 d and 21 d curing ages, the compressive strengths of N3 are 39.7 MPa and 57.85 MPa, respectively, representing increases of 39.54% and 6.83% compared to N1, indicating a significant enhancement in early strength. However, when the dosage further increases to 5%, excessive NA particles tend to agglomerate, forming weak interfaces and numerous pore defects within the matrix [19,33]. This leads to a reduction in the mechanical properties of the solidified body, with the final strength reaching only 50.60% of that of the blank group. From the three-dimensional contour plot in Figure 7b, it can be observed that all the NA-modified composite grouting materials exhibit a clear strength growth gradient from 1 d to 21 d. Especially under a 5% NA dosage, the ultrafine cement system demonstrates steady strength development. As shown in Figure 7c,d, the flexural strength of the solidified specimens follows a similar evolution trend to compressive strength with increasing NA content. The gradient changes in flexural strength contours on the projection plane are distinct. At a 5% dosage, the strength growth rate is most pronounced between 1 and 21 d, demonstrating excellent toughness development. Particularly, the toughening effect of NA particles is remarkable during the early hydration stage, with N3 showing a 55.41% increase compared to N1. This is primarily due to NA optimizing the pore structure of the matrix through particle filling and participating in hydration reactions to generate more cementitious products. These effects reduce inherent microcracks and effectively transfer and disperse bending stresses, thereby inhibiting crack initiation and propagation [14,25]. However, when the NA dosage reaches 5%, the 21 d flexural strength increases by only 5.89% compared to N1. This is because excessive agglomerates of NA particles and interface defects become stress concentration points, accelerating crack propagation under flexural loading and thereby degrading flexural performance. These results indicate that appropriate dosage of nano-Al_2_O_3_ has a significant enhancing effect on the mechanical strength for ultrafine cement grouting materials.

The NA dosage was selected by jointly considering workability, volume stability, and mechanical performance. Because reinforcement capacity is the primary function of the hardened grout, early-age strength was prioritized. Accordingly, 3 wt.% NA was recommended within the investigated range because it provided the highest early-age strength while maintaining acceptable fresh-state and volume stability characteristics.

**Figure 8:**
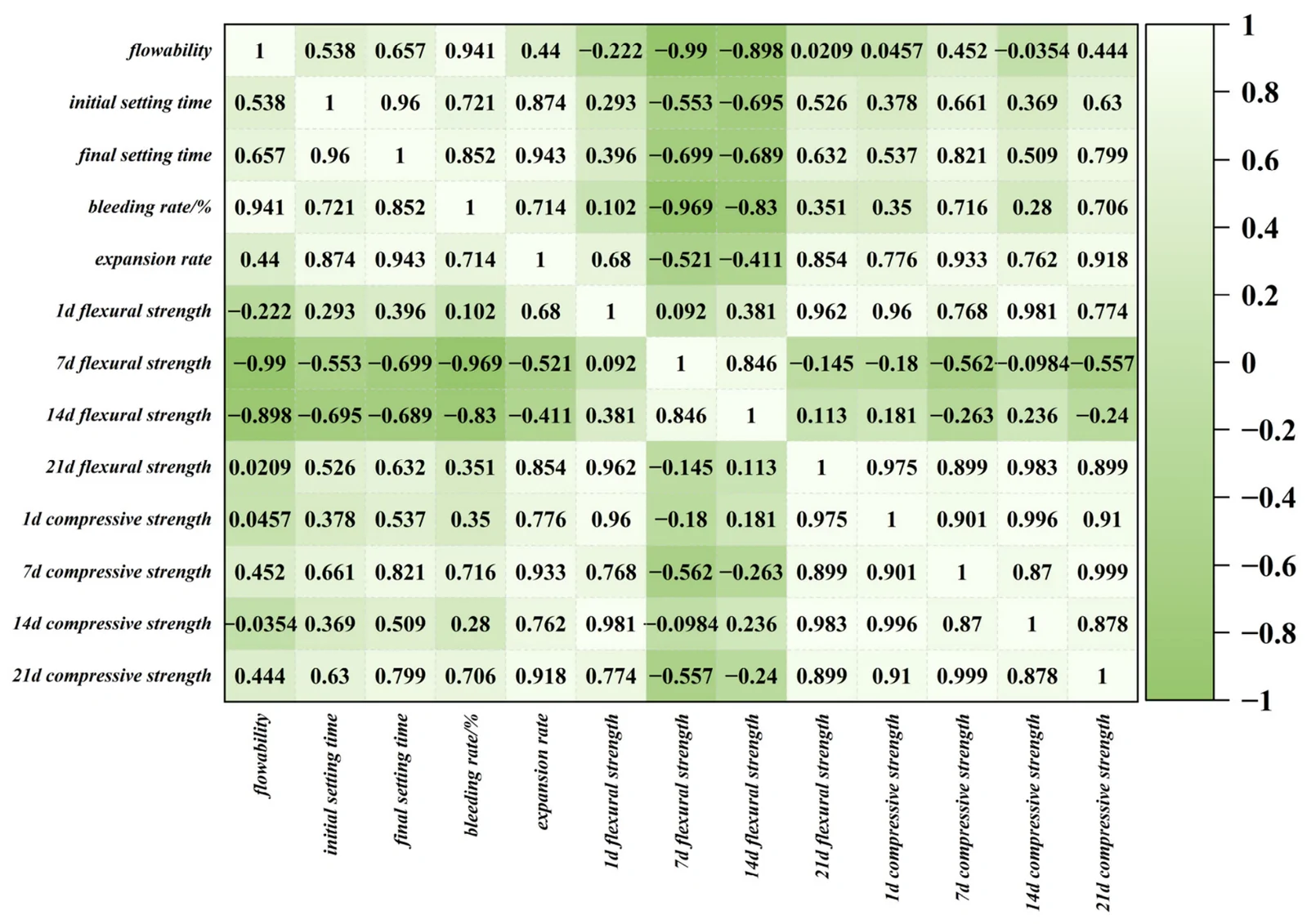
Pearson correlation coefficient matrix among key performance indicators.

Figure 8 shows the Pearson correlation coefficient matrix among key performance indicators of different NA-modified ultrafine cement composite grouting materials. The correlations of setting time and flowability with flexural and compressive strengths vary with curing age. Particularly, flowability exhibits strong negative correlations with the 7 d and 14 d flexural strengths, with Pearson correlation coefficients of −0.989 and −0.899, respectively. The expansion rate also shows a strong positive correlation with the 21 d flexural strength, with a correlation coefficient of 0.854. This indicates a positive association between expansion and long-term mechanical properties. Furthermore, several flexural and compressive strength indicators demonstrate strong correlations across different curing ages. Especially, the correlation between the 7 d and 21 d compressive strengths is the strongest, with a Pearson correlation coefficient of 0.999, indicating the consistent development of compressive performance during prolonged curing. The results suggest that, when optimizing the mix proportion of NA-modified ultrafine cement composite grouting materials, a systematic balance must be achieved among the interdependent relationships of flowability, expansion rate, and strength.

### 3.6. Microscopic Properties

**Figure 9:**
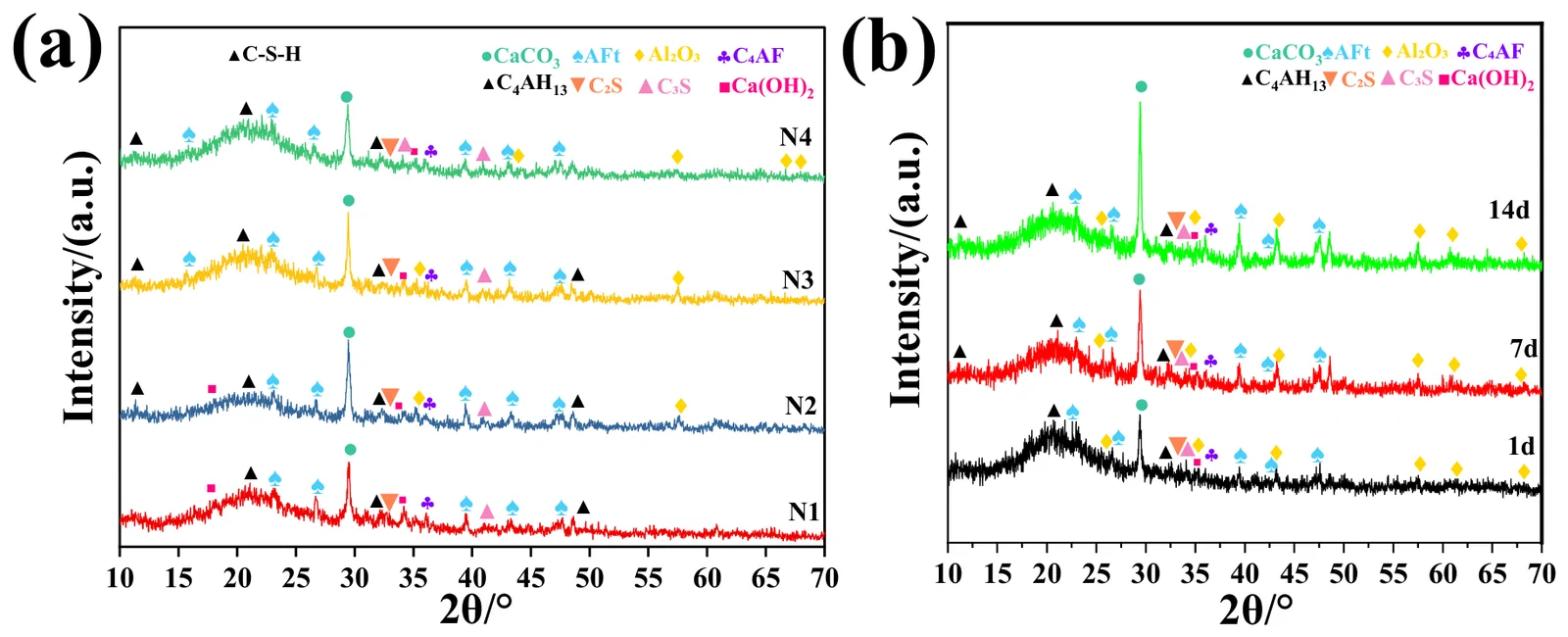
XRD pattern of NA-modified superfine cement composite grouting materials: (**a**) N1–4 (21 d); (**b**) N3 (1 d to 14 d).

To reveal the relationship between the mechanical properties and microstructural evolution of composite grouting materials, XRD, FTIR, SEM, and heat of hydration characterization techniques were employed to analyze the phase composition, chemical structure, and micro-morphology of the hydration products. Figure 9a shows the XRD patterns of the four types of solidified bodies at a curing age of 21 d. The main hydration products of the composite grouting material include ettringite (AFt), calcium silicate hydrate (C-S-H) gel, and calcium hydroxide (Ca(OH)_2_). The characteristic diffraction peaks of calcium carbonate (CaCO_3_) mainly originate from the carbonation of Ca(OH)_2_ on the surface. As the NA content increases, the diffraction peak intensity of NA gradually increases, while the intensities of the AFt and CaCO_3_ peaks initially rise and then decrease. When the NA content reaches 3%, the N3 solidified specimen exhibits the highest diffraction peak intensity. It indicates that an appropriate amount of NA effectively promotes the formation and crystallization of the primary hydration products in ultrafine cement. To further understand the hydration kinetics of N3, continuous XRD monitoring was conducted over the period from 1 to 14 d (Figure 9b). With increasing curing time, the diffraction peak intensities of CaCO_3_, C-S-H gel, and AFt continuously increase, reflecting ongoing hydration reactions. At 14 d, the significant improvement in AFt crystallinity coincides closely with the rapid growth stage of macroscopic mechanical strength, indicating that this period is critical for the strength development of the material.

**Figure 10:**
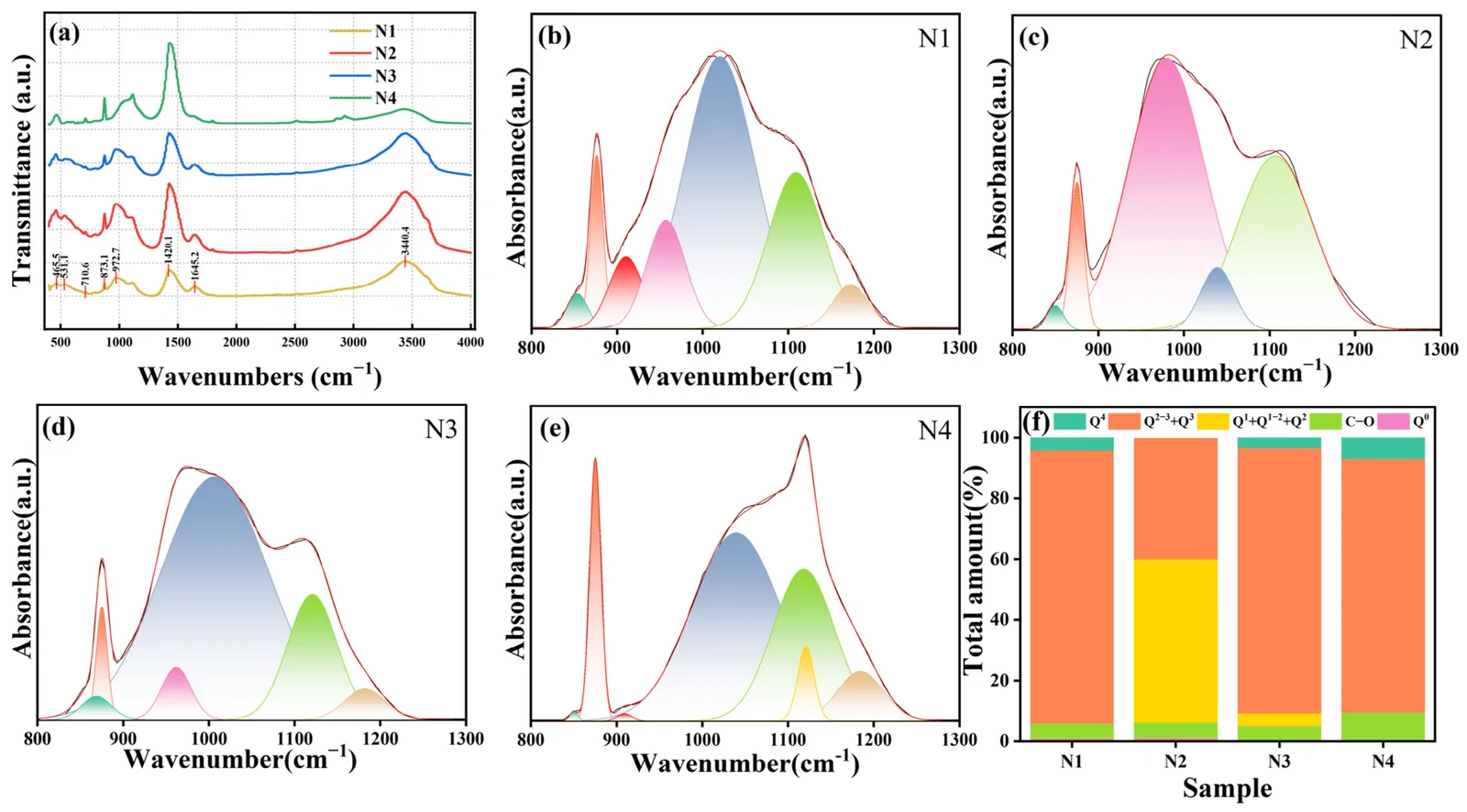
(**a**) FTIR spectra, (**b**–**e**) deconvolution fitting plots and (**f**) content of Q species in silicate tetrahedra of NA-modified superfine cement composite grouting materials.

Figure 10 presents FTIR spectra and deconvolution fitting images of five grouting materials cured for 21 d. As shown in Figure 10a, the absorption peaks at 465.5 cm^−1^ and 531.1 cm^−1^ originate from the bending vibrations of Si–O and Al–O bonds [34]. The peaks at 710.6 cm^−1^, 873.1 cm^−1^, and 1420.1 cm^−1^ correspond to carbonate ions formed during carbonation [35]. The main peak near 972.7 cm^−1^ arises from the characteristic vibration of the Si–O bond in C–S–H gel [36]. The strong broad peak near 1644.5 cm^−1^ is attributed to the O–H bending vibrations of adsorbed or bound water. The broad absorption band at 3440.4 cm^−1^ corresponds to the O–H stretching vibrations of hydrated products Ca(OH)_2_ and bound water. It indicates abundant hydroxyl functional groups within the system and their complex interactions with cement hydration products. Deconvolution and fitting of the spectral region between 800 cm^−1^ and 1300 cm^−1^ provide quantitative information on the structural evolution of hydration products, as shown in Figure 10b–e. In this range, the absorption peak at 850 cm^−1^ corresponds to unhydrated cement particles (Q^0^). The peak at 873.1 cm^−1^ is associated with C–O bond vibrations. The characteristic peaks at 910.7 cm^−1^ and 957.6 cm^−1^ are assigned to the Q^1–2^v_3_ and Q^2^v_3_ vibrational modes, respectively; those at 1022.9 cm^−1^ and 1039.9 cm^−1^ correspond to the Q^2–3^v_3_ and Q^2^v_3_ modes, respectively. The peak at 1108.7 cm^−1^ is related to the Q^3^v_2_ and v_3_ vibrational modes, and the peak at 1172.9 cm^−1^ represents the Q^4^v_3_ mode [37,38,39]. This series of characteristic peaks suggests a structural evolution pathway of silicate (aluminate) tetrahedra from low to high polymerization degrees during ultrafine cement hydration. Figure 10f shows the content of Q species in silicate tetrahedra. The silicon–oxygen tetrahedral structures of C–S–H/C–A–H gels in the four grouting materials are primarily concentrated in the Q^1^ + Q^1–2^ + Q^2^ and Q^2–3^ + Q^3^ regions, with both accounting for over 83% in total. Notably, when the NA content is 3%, the proportion of Q^2–3^ + Q^3^ in sample N3 is closest to that of N1, and no Q^0^ structure is detected. This indicates that an appropriate amount of nano-Al_2_O_3_ promotes the continuous reaction of unhydrated cement particles while maintaining a gel network dominated by medium- to high-polymerization chain-like and branched structures. Furthermore, N3 contains 4.15% of Q^1^ + Q^1–2^ + Q^2^ and 3.33% of Q^4^ structures, endowing the gel system with both segment growth capability and a certain degree of spatial crosslinking. This contributes to the formation of a uniform, continuous, and dense load-bearing network, thereby improving mechanical properties.

**Figure 11:**
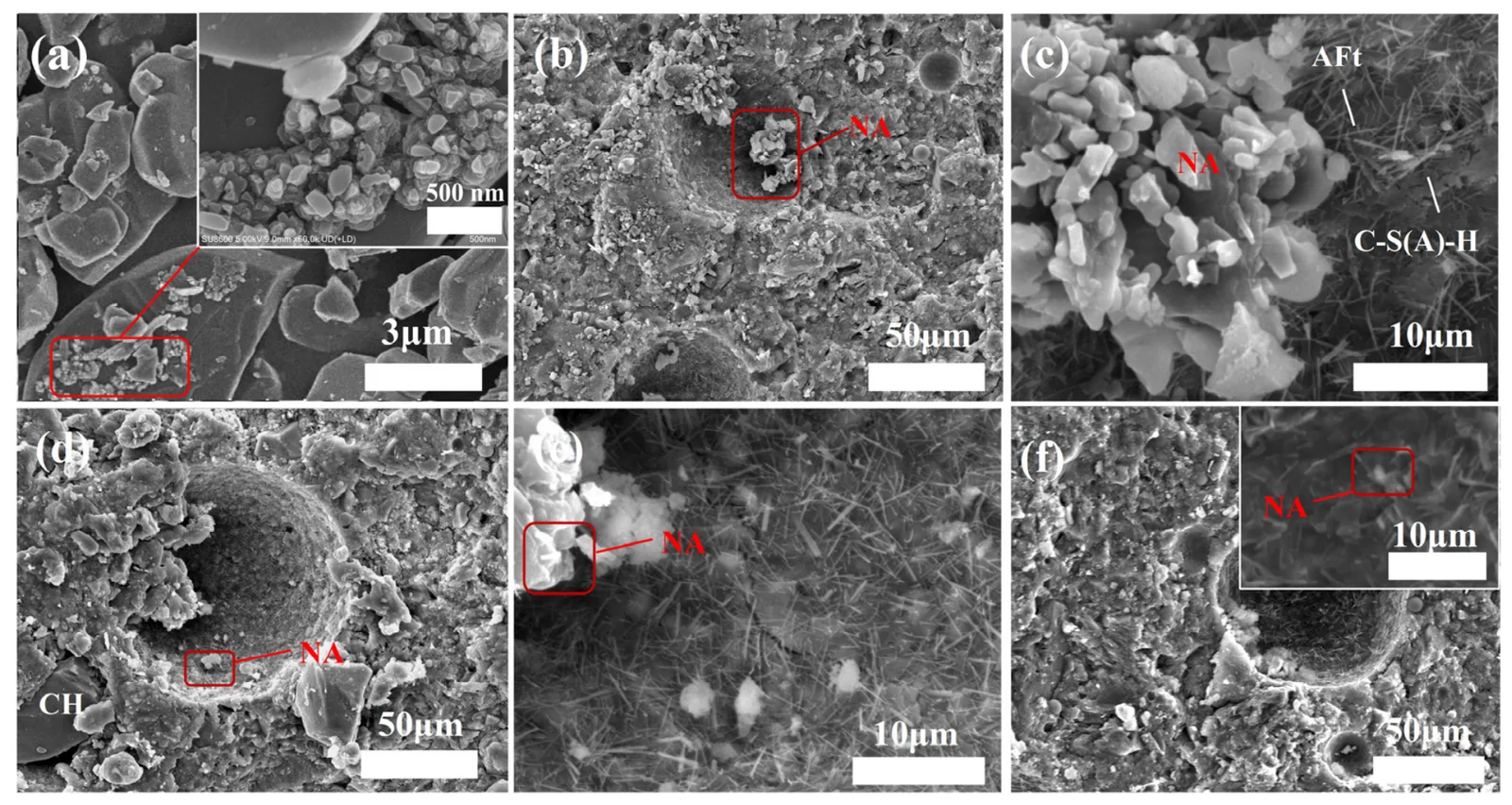
SEM images of N3 specimens at different ages: (**a**) nano-Al_2_O_3_; (**b**,**c**) 3 d; (**d**,**e**) 7 d; (**f**) 14 d.

Figure 11a shows the morphology of the Al_2_O_3_ powder. Micrometer-scale plate-like secondary structures are observed, while the enlarged image reveals abundant nanoscale particles attached to or clustered around these structures. Figure 11b–f present the microstructural evolution of the cross-sections of 3% Al_2_O_3_-modified N3 specimens at different curing ages. After 3 d of hydration, abundant needle-like AFt, flaky Ca(OH)_2_, and C–S(A)–H gel products have formed, with nano-Al_2_O_3_ particles clearly visible. However, the overall structure remains relatively loose, containing numerous pores of varying sizes. As the curing age increases to 7 d, cement hydration becomes increasingly complete, leading to further growth and interconnection of hydrated gel products. The pore structure undergoes reconstruction, and surface porosity is markedly reduced. With further extension of curing time (14 d), large amounts of hydrated products interpenetrate and grow together. A small number of Al_2_O_3_ particles adhere within the pores, forming a denser network structure with the matrix.

**Figure 12:**
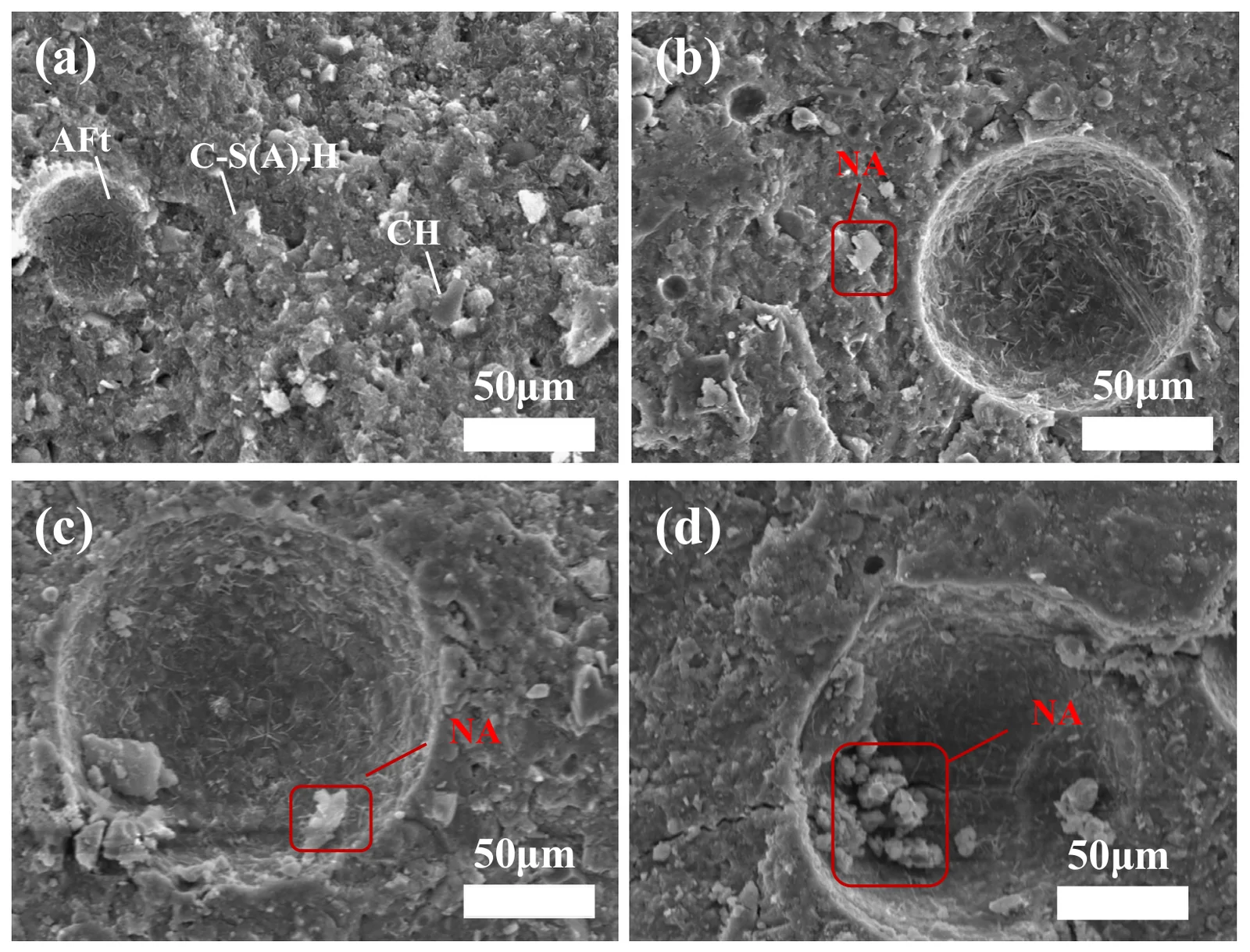
SEM images of NA-modified superfine cement composite grouting materials curing for 21 d: (**a**) N1; (**b**) N2; (**c**) N3; (**d**) N4.

Figure 12 shows the micro-morphology evolution of the cross-section of ultrafine cement grouting material solidified body with varying NA content. It is found that the amount of Al_2_O_3_ is markedly correlated with the composition of hydration products, microstructure, and density of the grouting material solidified body. At a curing age of 21 d, in the control group N1, AFt, Ca(OH)_2_, and C–S(A)–H hydration products have fully developed and interconnected to form a dense and network-like overall structure. However, the matrix contains numerous large pores and voids. With the addition of 1% NA, the pore size in specimen N2 decreases, and a small number of relatively well-dispersed Al_2_O_3_ particles are observed on the surface. When the NA content reaches 3%, the void structures are filled on the surface of N3, and Al_2_O_3_ nanoparticles couple with hydration products to form a highly compact structure. As the dosage increases further, particularly in N4, more Al_2_O_3_ particles agglomerate and accumulate on the surfaces of hydration products. Meanwhile, obvious pores and large cracks are observed within the matrix, which are unfavorable for mechanical performance development.

**Figure 13:**
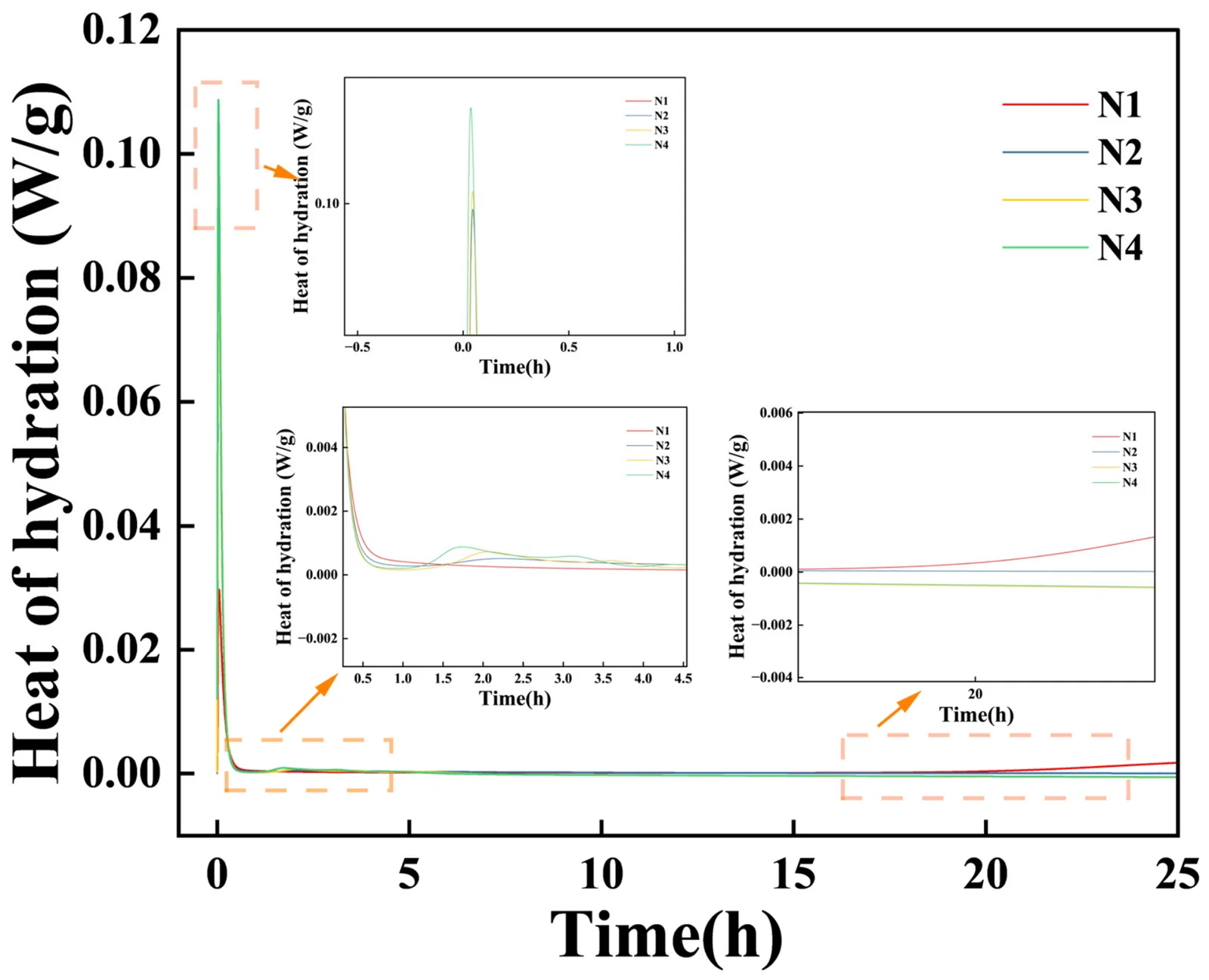
Hydration heat and cumulative heat curves of NA-modified ultrafine cement grouting materials.

Figure 13 shows the hydration heat and cumulative heat curves of the NA-modified ultrafine cement grouting materials. In the initial stage, as the NA content increases (N1 to N4), the peak value of the first exothermic peak rises. Particularly, N4 is close to 0.11 W/g, which is higher than N1. It suggests that the heterogeneous nucleation effect of Al_2_O_3_ accelerates the early dissolution of cement. The induction period shortens obviously with the increase in content, and the exothermic trough appears earlier. The hydration reaction enters the accelerated stage earlier. The second exothermic peak in the acceleration period increases earlier and rises to a higher peak with the increase in content. N4 has the highest peak, which shows that Al_2_O_3_ effectively promotes the hydration of C_3_S and the formation of hydrated calcium aluminate. After the deceleration period, the heat release rate of the high-content samples tends to be more stable earlier, while the low-content samples still have slow heat release in the later stage. For the cumulative hydration heat, it markedly increases in the early stage (0 to 24 h) with the increase in content. N4 has a cumulative heat much higher than N1. The difference in cumulative heat among different contents gradually narrows in the later stage. These results indicate that the doping of nano-Al_2_O_3_ on ultrafine cement has a significant strengthening effect on the early hydration, and excessive content will limit the later reaction due to agglomeration.

### 3.7. Mechanism Analysis

**Figure 14:**
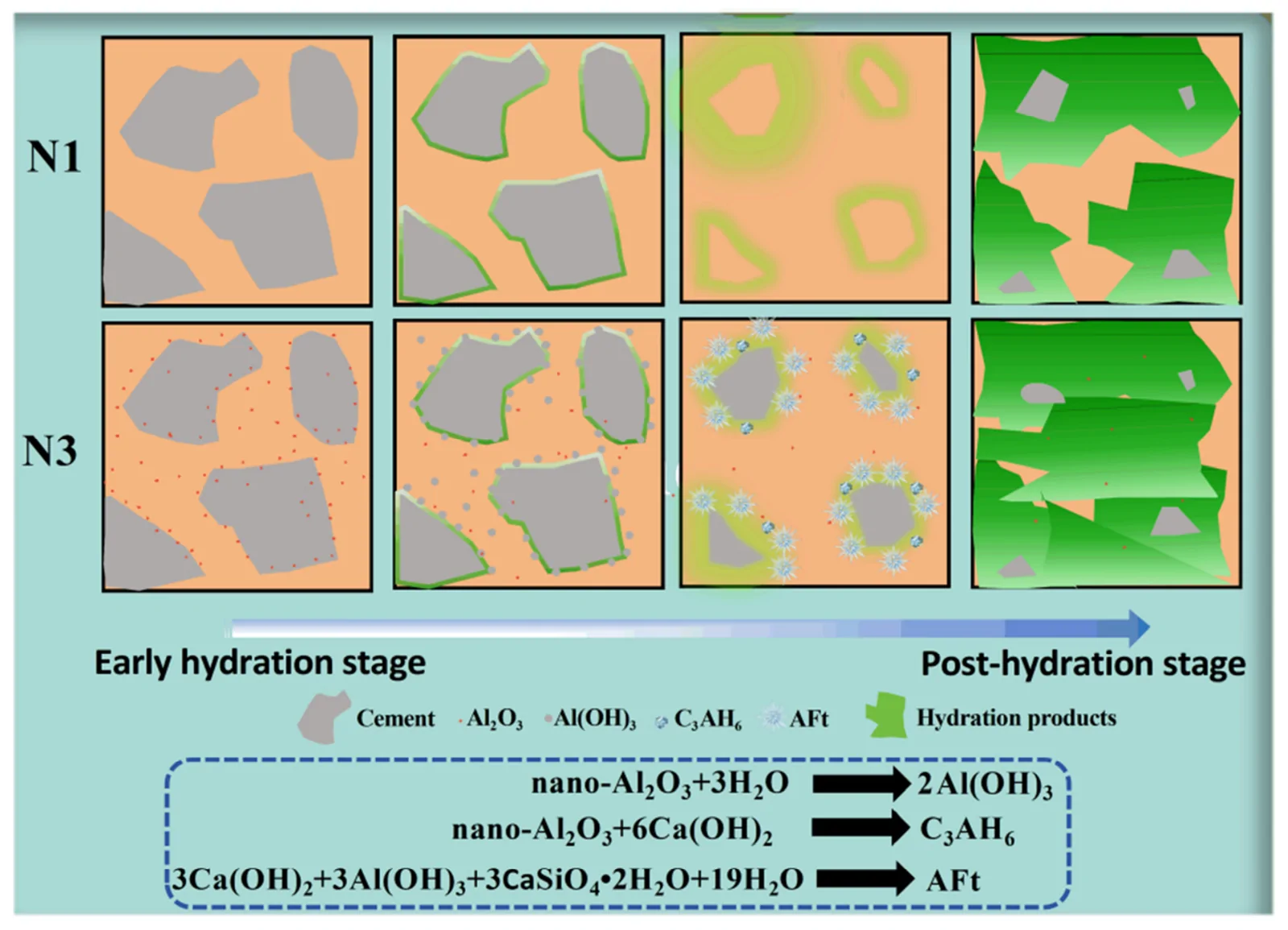
Modification mechanism of NA on superfine cement grouting materials.

Figure 14 illustrates the modification mechanism of Al_2_O_3_ on superfine cement grouting materials, which mainly manifests in three aspects. Firstly, Al_2_O_3_ nanoparticles with a large specific surface area and high surface activity can serve as heterogeneous nucleation sites for the hydration products and reduce the nucleation barrier between C-S-H gel and Ca(OH)_2_, markedly shortening the hydration-induced period. This accelerates the early hydration process, markedly enhancing the early heat release rate and strength development. Secondly, Al_2_O_3_ nanoparticles with certain pozzolanic activity can undergo a secondary hydration reaction with Ca(OH)_2_ can be generated during cement hydration, forming products such as calcium aluminate hydrate (AFt/AFm).It not only consumes easily leachable Ca(OH)_2_ but also fills capillary pores, refines pore diameters, and reduces porosity. This will optimize the microstructure of the grouting material, enhancing density and impermeability. Finally, the addition of fine plate-like NA modifies particle packing and interparticle interactions in the fresh slurry. These effects increase the resistance to segregation and water migration, thereby reducing bleeding. However, increasing the NA dosage also increases flow resistance, while particle agglomeration becomes more pronounced at 5 wt.%, which is unfavorable for workability and subsequent microstructural development. Therefore, such an outstanding mechanical property is mainly attributed to a triple coupling mechanism of the hydration regulation, microstructure and filling effect of superfine cement by Al_2_O_3_ nanomaterials in combination with multi-component admixtures.

## 4. Conclusions

(1) To optimize the working performance and mechanical characteristics of cement grouting materials, a new type of composite grouting material with high early strength and high toughness was successfully developed by introducing nano-Al_2_O_3_ into ultrafine cement in combination with multi-component admixtures.
(2) The NA content had a significant regulatory effect on the working performance of the composite grouting material. As the content of NA increased, the flowability of the paste decreased, and the bleeding rate reduced. However, the setting time and volume expansion rate showed a trend of increasing first and then decreasing. The N3 specimen with 3% NA had the highest comprehensive performance, with good micro-expansion characteristics and shortened initial setting time, which was 12.5% lower than that of the blank group.
(3) The mechanical property enhancement by NA showed a threshold effect for the composite grouting material. The compressive and flexural strengths increased first and then decreased with the increase in NA content. Compared with the control group, the N3 specimen with 3% showed an early compressive strength increase of 39.54% and a 55.41% increase in 1 d flexural strength, demonstrating excellent toughening effects.
(4) Microscopic characterizations confirmed that the doping of NA promoted the early hydration heat of ultrafine cement, shortened the induction period, and accelerated the hydration process. In addition, the active NA can consume Ca(OH)_2_ to generate more hydrated calcium aluminate gel products, resulting in an improvement in the compactness of the cementitious matrix. Such an outstanding mechanical property was mainly attributed to a triple coupling mechanism of the hydration regulation, microstructure and filling effect of superfine cement by Al_2_O_3_ nanomaterials in combination with multi-component admixtures.

## Figures and Tables

**Table 1:** Comparison of representative Al_2_O_3_-modified cementitious systems.

Study	Material System	Al_2_O_3_ Characteristic Parameters	Main Evaluation and Findings
Zhou et al. [17]	Ordinary Portland cement paste	γ-Al_2_O_3_, 5 wt.%	Early hydration and phase evolution were examined. γ-Al_2_O_3_ promoted early AFt formation and the C_3_A reaction.
Yu et al. [16]	Ordinary Portland cement paste	α- and γ-Al_2_O_3_, both at 5 wt.%	Sulfate durability was evaluated. Both phases increased long-term strength loss and expansion, with greater deterioration caused by γ-Al_2_O_3_.
Iqbal et al. [13]	Cement paste containing a modified fly ash carrier	In situ-grown and commercial α-/γ-Al_2_O_3_, 0.5–1.5 wt.%	Workability, rheology, hydration, strength, and microstructure were evaluated. In situ-grown Al_2_O_3_ showed better dispersion and performance.
Zhou et al. [18]	CSA–Portland cement–fly ash–slag rapid-setting grout	Nano-Al_2_O_3_, 0.4–2.0 wt.%	Flowability, strength, and microstructure were evaluated. The favorable performance occurred at 0.4 wt.%, with no further improvement at higher dosages.
Wu et al. [19]	Cement–fly ash grouting material	Nano-Al_2_O_3_, 1–4 wt.%; crystal phase not specified	Strength and microstructure were evaluated. Strength peaked at 1 wt.% and declined at 2–4 wt.%.
This study	Multi-component ultrafine cement grouting material	α-Al_2_O_3_, 0, 1, 3, and 5 wt.%	Flowability, bleeding, setting, expansion, strength, hydration heat, phase composition, chemical structure, and morphology were evaluated using XRD, FTIR, and SEM.

**Table 2:** Mix proportion of grouting material (wt.%).

Sample	Ultrafine Cement	Expansion Agent	Accelerator	NA	Superplasticizer	Water
N1	100	4.5	3.5	0	0.35	35
N2	100	4.5	3.5	1	0.35	35
N3	100	4.5	3.5	3	0.35	35
N4	100	4.5	3.5	5	0.35	35

## Data Availability

The data that support the findings of this study are available from the corresponding author upon reasonable request.
